# Evaluation of first-trimester neutrophil-lymphocyte ratio and platelet-lymphocyte ratio values in pregnancies complicated by intrauterine growth retardation

**DOI:** 10.4274/tjod.galenos.2020.81592

**Published:** 2020-07-29

**Authors:** Harun Egemen Tolunay, Hasan Eroğlu, Erol Nadi Varlı, Mustafa Akşar, Dilek Şahin, Aykan Yücel

**Affiliations:** 1 University of Health Sciences Turkey, Etlik Zübeyde Hanım Maternity and Women’s Health Teaching and Research Hospital, Ankara, Turkey

**Keywords:** Intrauterine fetal growth retardation, neutrophil-lymphocyte ratio, platelet-lymphocyte ratio

## Abstract

**Objective:**

The objective of this study is to compare the first-trimester hemogram parameters [neutrophil-lymphocyte ratio (NLR) and platelet-lymphocyte ratio (PLR)] of pregnant women complicated by intrauterine growth retardation (IUGR) and normal pregnant women.

**Materials and Methods:**

We retrospectively evaluated the medical records of pregnant women (n=50) complicated with IUGR and pregnant women in the control group (n=50).

**Results:**

The first-trimester NLR and PLR values of the pregnant women complicated by IUGR were 6.59±1.12 and 117.2±16.00, respectively. The first-trimester NLR and PLR values of the pregnant women in the control group were 2.84±0.55 and 112.80±13.01, respectively. There was a statistically significant difference between the two groups with respect to NLR (p<0.001).

**Conclusion:**

Pregnancies complicated by IUGR have high neonatal mortality and morbidity rates. Therefore, the early diagnosis of disease and appropriate management are extremely crucial for both fetal and maternal prognoses. High NLR values in the first trimester may contribute to the early diagnosis of IUGR.

**PRECIS:** Harun Egemen Tolunay’s article, “Evaluation of first-trimester neutrophil-lymphocyte ratio and platelet-lymphocyte ratio values in pregnancies complicated by intrauterine growth retardation (2020)”, analyses the predictivity of neutrophil-lymphocyte ratio and platelet-lymphocyte ratio in intrauterine growth retardation.

## Introduction

Intrauterine growth retardation (IUGR) occurs when the fetus fails to reach its growth potential because of genetic and environmental factors. Sonography-based diagnosis defines IUGR as the condition in which the fetal weight is below the tenth percentile. IUGR is the most common cause of perinatal deaths after prematurity. Studies have reported that IUGR is observed in nearly 7-10% of all the pregnancies. There has been an increased risk of neonatal morbidity and mortality in pregnancies complicated by IUGR. The diagnosis of IUGR is usually based upon ultrasonographic assessment^(1,2,3)^.

One of the most important objectives of obstetric follow-up is to identify the patients at risk for perinatal problems. The early recognition of this disease and early treatment interventions are very crucial in reducing the rates of morbidity and mortality in IUGR. Moreover, the diagnosis of disease and appropriate management are extremely important for both fetal and maternal prognoses^(4,5)^.

Complete blood count [(CBC) or hemogram] is a frequently used basic laboratory test. White blood cell (WBC) count, red blood cell count, and platelet counts are some of the parameters used in this simple test. As an inexpensive and widely available marker in clinical usage, neutrophil-lymphocyte ratio (NLR) and platelet-lymphocyte ratio (PLR) have been proposed in the different areas of obstetrics and gynecology medical practice^(6,7,8,9)^.

The purpose of this study is to evaluate the first-trimester NLR and PLR values in pregnant women complicated by IUGR and pregnant women of the control group. This study also evaluates fetal growth in both these groups.

## Materials and Methods

We retrospectively reviewed the medical records of the study participants in the University of Health Sciences Turkey, Etlik Zübeyde Hanım Maternity and Women’s Health Teaching and Research Hospital, Clinic of Perinatology. We included a total of 100 patients (50 pregnant women with IUGR and 50 healthy pregnant women) who were admitted to our hospital. Fetuses with an estimated fetal weight of less than tenth percentile in the ultrasonographic evaluation were diagnosed with IUGR. We randomly selected the control group from the healthy pregnant women who did not had any maternal-fetal conditions. The Local Ethics Committee University of Health Sciences Turkey, Etlik Zübeyde Hanım Maternity and Women’s Health Teaching and Research Hospital granted its approval for the conduct, protocol, and procedures of the study (approval number: 01-20-1).

We examined the first-trimester routine hemogram parameters of the participating women. NLR was calculated by dividing the absolute neutrophil count with the absolute lymphocyte count, whereas PLR was determined by dividing the absolute platelet count with the absolute lymphocyte count. We compared the first-trimester hemogram parameters (NLR and PLR) of both the groups.

### Statistical Analysis

We used the Statistical Package for the Social Sciences version 20.0 for Windows for all the statistical analyses of this study. Importantly, we preferred the non-parametric tests according to the tests of normality results. We used the Mann-Whitney U test to compare the continuous variables. P-value of less than 0.05 was considered as statistically significant for this study.

## Results

The mean ages of the pregnant women with IUGR and pregnant women in the control group were 28±2.02 and 28.52±2.69 years, respectively. The mean numbers of gravida in the IUGR and control groups were 2.62±0.72 and 2.54±1.16, respectively. The mean parity numbers in the IUGR and control groups were 1.54±0.50 and 1.58±0.81, respectively. The mean gestational weeks in the IUGR and control groups were 36.04±1.12 and 36.08±1.38, respectively. The first-trimester NLR and PLR values of the pregnant women complicated by IUGR were 6.59±1.12 and 117.2±16.00, respectively. The first-trimester NLR and PLR values of the pregnant women in the control group were 2.84±0.55 and 112.80±13.01, respectively. There was a statistically significantly difference between the two groups with respect to NLR (p<0.001) (Table 1).

## Discussion

This is the first study, to our knowledge, which examined the first-trimester hemogram parameters in pregnant women complicated with IUGR. We hypothesized that the inflammatory process during pregnancy can help in the diagnosis of IUGR. We showed that the first-trimester NLR and PLR values are higher in the IUGR group as compared to the control group. Importantly, the NLR values were statistically significantly higher in the IUGR group as compared to the control group.

Various causes can be attributed to the manifestation of IUGR. These causes can be fetal, maternal, and placental factors. Most of the IUGR cases (especially recurrent IUGR) occur due to placental ischemia/inflammation. These placental problems may present as IUGR, ablation, and preeclampsia. These conditions may result in preterm birth and pregnancy loss.

IUGR scanning in the general obstetric populations are based on a broader determination of risk factors and physical assessment of fetal growth. After clinical suspicion, there should be a detailed evaluation of the fetus, placenta, and amniotic fluid. Early diagnosis is very crucial in the proper management of IUGR^(10,11)^.

NLR and PLR have been used as the inflammation markers in recent years. These parameters can be obtained quickly and cheaply from the CBC test. NLR and PLR are increasingly being used as the indicators of cancer and various systemic diseases as well as systemic inflammation. Researchers have recently studied the usage of CBC parameters in the field of obstetrics and perinatology. A recent research conducted by Örgül et al.^(12)^ had shown that increased first-trimester WBC and neutrophil counts may be predictive of early-onset preeclampsia. Although studies have been conducted to determine whether these parameters may have predictive values in the cases such as endometrioma and tubo-ovarian abscess in gynecology and obstetrics practice, there is no study that aimed to determine the value of these parameters in IUGR^(13,14)^.

Preterm birth, IUGR, and stillbirth have been strongly associated with antenatal inflammation. The authors reported that the maternal inflammation and organization of vascular beds, which are to be indicated by NLR, were associated with this poor pregnancy outcome and fetal development. Maternal systemic inflammation during pregnancy may restrict embryo-fetal growth. Consistent with our results, the literature data also showed that the increased maternal inflammatory response was accompanied by IUGR^(15)^.

High NLR and PLR values in the first trimester during pregnancy may be an important and predictive biomarker of impaired intrauterine growth. High NLR and PLR values in the first trimester appear to reflect an increased inflammation. Increased placental inflammation in the etiology of IUGR may support this condition. Our study shows that increased NLR and PLR values in the first trimester may predict IUGR. The limitations of our study are as follows: The absence of placental examination, low number of pregnant women, and the lack of newborn outcomes.

## Conclusion

The clinical usage of NLR and PLR in hemogram tests may facilitate the diagnostic process of IUGR pregnancies. These parameters in the first trimester of pregnancy may serve as an important biomarker in the diagnosis of IUGR.

## Figures and Tables

**Table 1:**
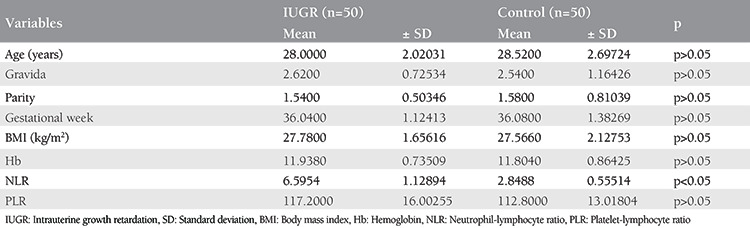
Characteristics of the patients
